# Survival, early growth and impact of damage by late-spring frost and winter desiccation on Douglas-fir seedlings in southern Sweden

**DOI:** 10.1007/s11056-018-9635-7

**Published:** 2018-03-08

**Authors:** Cecilia Malmqvist, Kristina Wallertz, Ulf Johansson

**Affiliations:** 1 Department of Forestry and Wood Technology, Faculty of Technology, Linnaeus University, 351 95 Växjö, Sweden; 2 Asa Forest Research Station, Swedish University of Agricultural Sciences, 360 30 Lammhult, Sweden; 3 Tönnersjöheden Experimental Forest, Swedish University of Agricultural Sciences, P.O. Box 17, 310 38 Simlångsdalen, Sweden

**Keywords:** Climate change, *Pseudotsuga menziesii*, Non-native species

## Abstract

Introduction of non-native species, such as Douglas-fir (*Pseudotsuga menziesii* (Mirb.) Franco), can be a means of mitigating the effects of climate change by meeting the growing demand for biomass and high quality wood. The aim of this study was to investigate early growth, survival and damage from late-spring frost and winter desiccation. A provenance trial with four coastal and three interior provenances of Douglas-fir originating from British Columbia, Canada, was established in Southwest Sweden (56°43′N, 13°08′E). Seedling height, length of the leading shoot, and occurrence of frost damage, were measured after one, three, and six growing seasons. Timing of bud break in spring was also observed. The interior Douglas-fir were more frequently damaged by late-spring frost compared to the coastal Douglas-fir. The interior Douglas-fir still had a higher survival after six growing seasons compared to the coastal variety. All provenances were damaged by winter desiccation, but the provenances originating from the coastal area were more severely damaged. Choice of variety may reduce the risk for either late-spring frost or winter desiccation.

## Introduction

Climate change provides new challenges to Nordic forestry (IPCC 2014). Use of non-native species, such as Douglas-fir (*Pseudotsuga menziesii* (Mirb.) Franco), can be a means of mitigating the effects of climate change and meeting the growing demand for biomass (Werner et al. 2010; Lundmark et al. 2014). Interest in Douglas-fir has increased in Sweden because it provides commercially valuable wood (Hermann and Lavender 1999; Hansson 2014). It is also attractive due to potentially high yield (Karlberg 1961; Nord-Larsen et al. 2009), wide site adaptability, and capacity to adapt to changing environmental conditions (Isaac-Renton et al. 2014). Ever since Douglas-fir was introduced to Europe, choice of suitable provenances has been an important question. The coastal Douglas-fir variety (*P. menziesii* var. *menziesii*) has shown higher growth potential than the interior Douglas-fir variety (*P. menziesii* var. *glauca*) in many European provenance trials (Veen 1951; Lundberg 1957; Kleinschmit et al. 1974; Larsen and Kromann 1983; Kleinschmit and Bastien 1992; Cafourek 2001; Konnert and Ruetz 2006; Petkova et al. 2008, 2014). Hybrids between the coastal and interior varieties have shown good performance in Saxony, Germany (Braun 1999). In Norway, Finland and Sweden, provenance trials found the interior variety to be superior to the coastal variety (Kurkela 1981; Magnesen 1987; Martinsson and Kollenmark 2001). In northern Sweden, only the most northern provenances of interior Douglas-fir survived (Martinsson and Kollenmark 2001).

Susceptibility to frost is one of the important traits that differ among provenances (Larsen 1978; Snäll 2000; O’Neill et al. 2000, 2001; Hansen 2007). Frost tolerance, along with growth cessation and bud formation, is affected by a combination of environmental factors, i.e. photoperiod length and air and soil temperature (Wareing 1956; Ekberg et al. 1979; Lavender 1981; Dormling 1993; Hannerz 1999; Hannerz and Westin 2000). Frost damage can be subjected directly to vegetative buds, stems, shoots, foliage, and roots or indirectly by desiccation, photoinjury and photoinhibation. Frost damage varies not only with the severity of frost relative to plant hardiness but also with respect to plant size and health (Krasowski and Simpson 2001). In the Nordic countries, frost events can occur during almost any time of the year.

Winter desiccation occurs under conditions of low night temperature and frozen soil combined with very high irradiation and low wind speed (Christersson and von Fircks 1988). Symptoms of winter desiccation include red foliar discoloration and root death. Livingston (1995) suggests that damage during winter results from a complex interaction involving minimum temperature, number of freeze and thaw cycles, cooling rate, and thawing rate. Sakai and Larcher (1987) emphasize the combination of large short-term fluctuations of shoot and soil surface temperatures on sunny sites as a cause of damage.

The objective of the present study was to compare early growth and survival and effects of late-spring frost and winter desiccation among seven Douglas-fir provenances in southern Sweden.

## Materials and methods

**Fig. 1:**
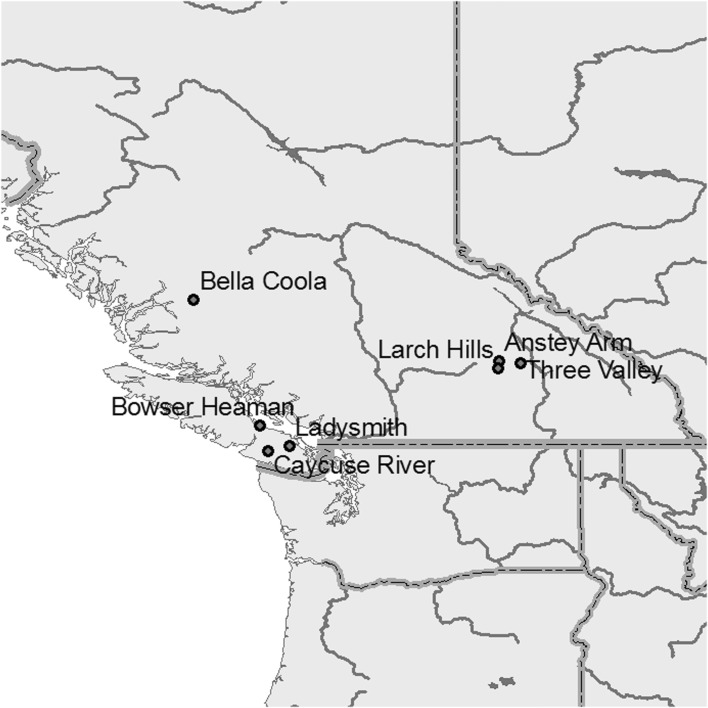
Map of the origin of the provenances in British Columbia, Canada

Seven provenances of Douglas-fir from British Columbia, Canada (Table 1, Fig. 1), were used in the trial at Tönnersjöheden Experimental Forest, Southwest Sweden (lat. 56°43′N, long. 13°08′E, alt. 75 m a.s.l.).

**Table 1:** The origin of Douglas-fir seeds collected in British Columbia, Canada, and used in this study

Provenance	Latitude	Longitude	Altitude	Variety
Caycuse River	48°50′N	124°29′W	550 m	Coastal^a^
Ladysmith	48°57′N	123°58′W	549 m	Coastal^a^
Bowser Heaman	49°26′N	124°41′W	Unknown	Coastal^b^
Bella Coola	52°25′N	126°15′W	150 m	Coastal^a^
Three Valley	50°55′N	118°27′W	710 m	Interior^a^
Anstey Arm	50°58′N	118°58′W	610 m	Interior^a^
Larch Hills	50°48′	119°00′W	670 m	Interior^a^

^a^Seeds collected in stands

^b^Seed orchard

The experimental site was harrowed in April 2009, 2 m between the rows, and planted in May 2009 with 2-year old bare-rooted hybrid larch (*Larix * × *eurolepis* Henry) seedlings (seed orchard Maglehem) and in May 2010 with 2-year old containerized seedlings of Douglas-fir. The seedlings were planted in alternate rows of Douglas-fir and hybrid larch, one row of Douglas-fir followed by one row of hybrid larch. The Douglas-fir seedlings were grown in a commercial nursery, stored in a freezer during the winter prior to planting and kept in a cooler during the spring prior to planting. The spacing in plantation was 2.0 × 4.0 m for Douglas-fir and 2.5 × 4.0 m for hybrid larch. The main reasons for mixing the Douglas-fir with hybrid larch was to create a frost shelter for the Douglas-fir seedlings and to avoid large gaps in the stand if many of the Douglas-fir seedlings died. The larch seedlings were considered to influence all provenances of Douglas-fir in the same way because the distance between the larch and Douglas-fir seedlings were the same all over the site. Therefore, the larch seedlings were not further considered in the analyses. Height of seedlings were recorded as soon as the seedlings were planted: Caycuse River 33.1 cm, Ladysmith 29.0 cm, Bowser Heaman 38.2 cm, Bella Coola 35.7 cm, Three Valley 30.7 cm, Anstey Arm 35.4 cm, and Larch Hills 33.9 cm. Mean height for all seedlings was 33.7 cm. The seedlings were all treated with insecticides (Merit Forest WG, active ingredient: imidakloprid) immediately after planting to prevent damage by pine weevils (*Hylobius abietis*).

The experiment had a randomized block design. In each of three blocks, seven plots were randomly placed, one for each provenance. The plot size was 40 × 40 m. In each plot, 36 seedlings were measured, making 108 measured seedlings per provenance and 756 measured seedlings in total for the experiment.

Seedling height and length of the leading shoot were measured after one, three, and six growing seasons. Damage, due to pine weevils, fungi, frost, drought, competing vegetation, wild animals, insects (other than pine weevils), and unknown reasons, were noted on the same occasions. Browsing, horn sweeping and gnawing from roe deer, hare and rodents were all classified as damage due to wild animals. Damage due to frost was divided into a) damage from late-spring frost and b) damage from winter desiccation. Symptom of damage from late-spring frost was characterized by damage to the annual shoots. First, they lost their turgor and later on became brown-coloured. Damage from winter desiccation was characterized by red foliar discoloration in early spring. Damage from drought would have similar symptoms, but during summer or early autumn, not in early spring. In addition, observations of late-spring frost damage and timing of bud burst were made during spring 2011, and damage by winter desiccation was made during spring 2013. The degree of damage was classified as 0 = undamaged, 1 = insignificantly damaged, 2 = slightly damaged, 3 = heavily damaged, 4 = life-threatening damaged, and 5 = dead.

**Fig. 2:**
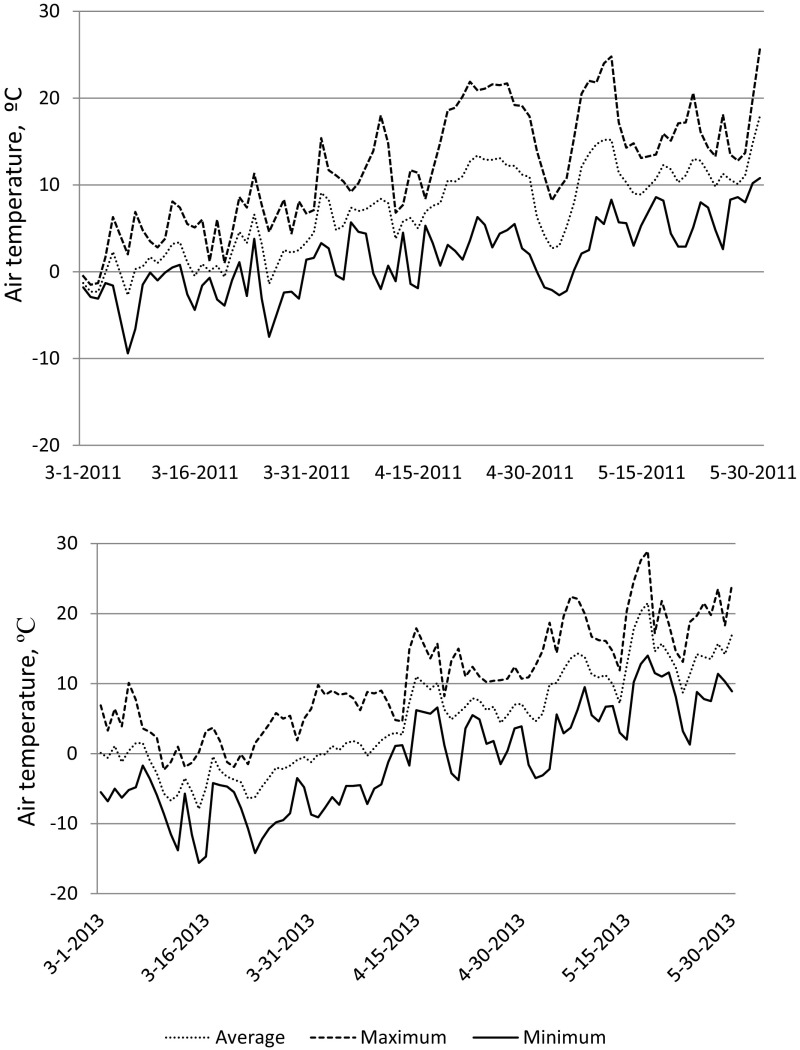
Air temperatures with daily average, maximum, and minimum values recorded 170 cm above ground from March 1 to May 31, 2011 (above) and 2013 (lower graph)

The annual precipitation in the area, 1057 mm, and mean annual temperature, 6.4 °C was calculated as annual mean between 1961 and 1990 (Alexandersson and Eggertsson Carlström 2001). In the early years of the trial (2010–2014), annual precipitation was 1094, 1181, 1297, 951 and 1268 mm respectively, of which approximately 70% was received during the growing season (Ottosson Löfvenius 2011, 2012, 2013, 2014, 2015). Air temperature during the last week of April 2011 was high for that time of the year. For 6 days in a row, air temperatures exceeded 20 °C for more than 4 h. This warm period was followed by a period of low temperature. During the first week of May 2011, freezing temperatures, lasting more than 6 h, occurred four times (Fig. 2). In 2013, the climatic conditions during March and April were characterized by very low night temperatures, and large differences between day and night temperatures (Fig. 2).

In order to estimate the possibility that the frost damage, caused by late-spring frost in 2011, interfered with the amount and degree of damage caused by winter desiccation in 2013, two sets of cross-tables were evaluated (Table 2).

**Table 2:**
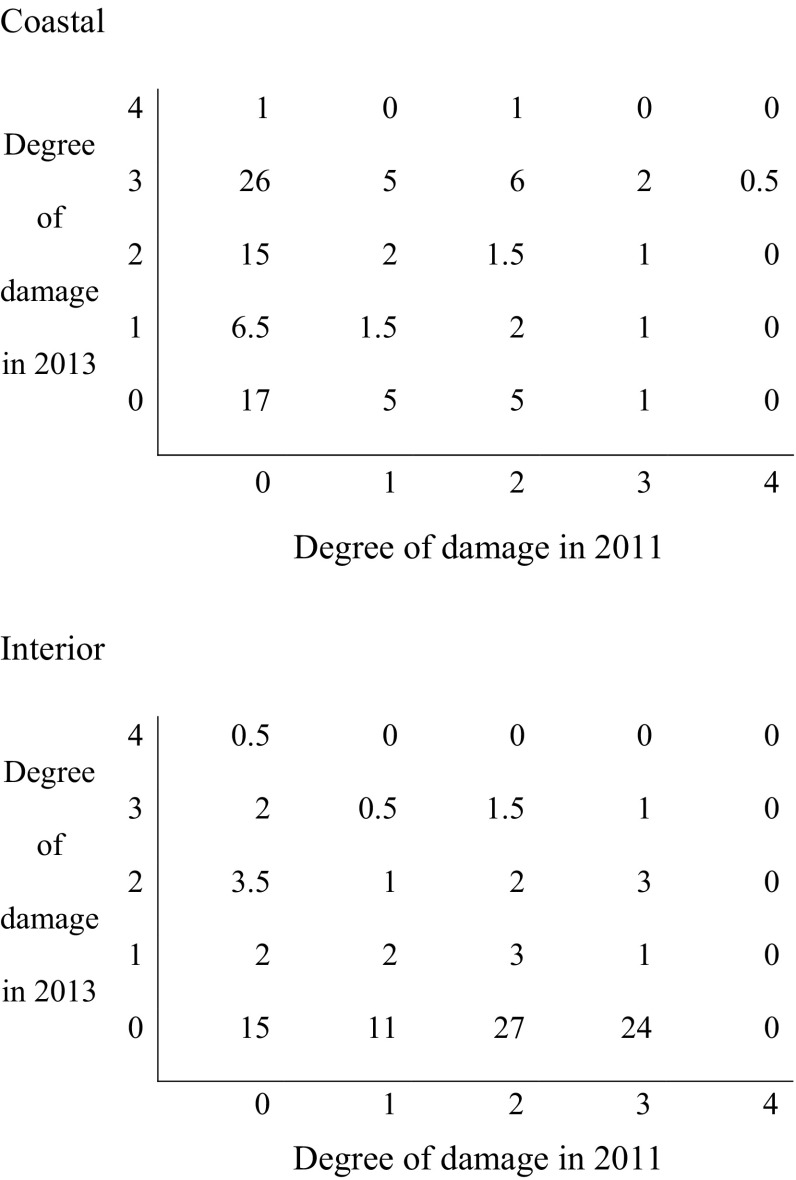
Proportion (%) of seedlings in each damage class from late-spring frost in 2011 and winter desiccation in 2013

The degree of damage was classified as 0 = undamaged, 1 = insignificantly damaged, 2 = slightly damaged, 3 = heavily damaged, 4 = life-threatening damaged. The upper table relates to the coastal variety and the lower table relates to the interior variety

### Statistical analyses

SAS software (SAS Institute, Cary, NC, USA) was used for all data analyses. Mean values for height and length of leading shoot were calculated for each block and provenance before any analyses were performed. The experiment was treated as a randomized block design with block as random factor, using the following (PROC MIXED SAS) model:

$$Y_{ij} = \mu + \alpha_{i} + \beta_{j} + \varepsilon_{ij}$$

where μ is the overall mean, α_i_ the block effect (i = 1–3), β_j_ the provenance effect (j = 1–7) and ε_ij_ the experimental error. Data for seedling survival and damage were analysed with generalised linear models in PROC GLIMMIX in SAS 9.4. When significant differences were identified (*p* < 0.05), Turkey’s tests were used to separate effects of individual factors. The same model was used to compare the varieties instead of provenances. Differences between means were deemed significant at α = 0.05.

## Results

### Survival

**Fig. 3:**
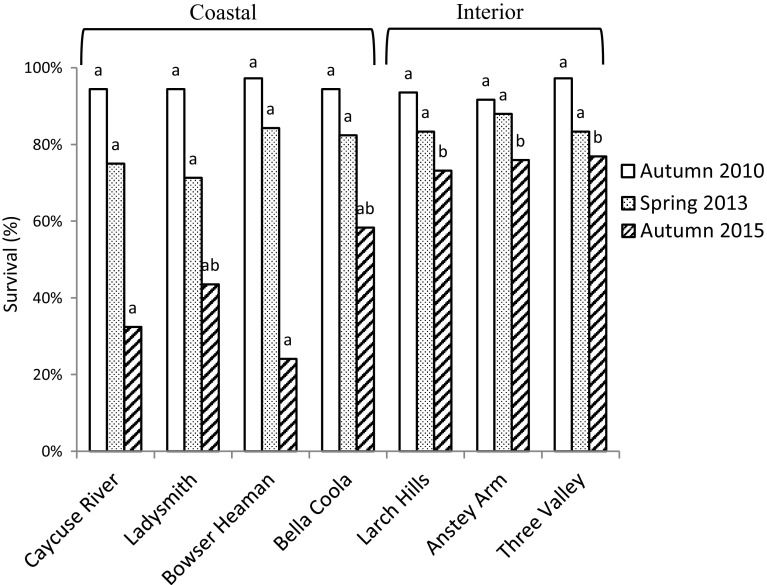
Survival (%) after one (Autumn 2010), three (Spring 2013) and six (Autumn 2015) growing seasons. Means with the same letter indicate no significant differences at *p *= 0.05

After one and three growing seasons survival did not differ significantly among the individual provenances (Autumn 2010: *p* = 0.83, Spring 2013: *p* = 0.76) or when grouped by coastal or interior varities (Autumn 2010: *p* = 0.77, Spring 2013: *p* = 0.31). After six growing seasons, however many of the coastal Douglas-fir seedlings had died and a significant difference in survival rate was observed among individual provenances (*p* = 0.030) (Fig. 3).

### Frost damage

**Fig. 4:**
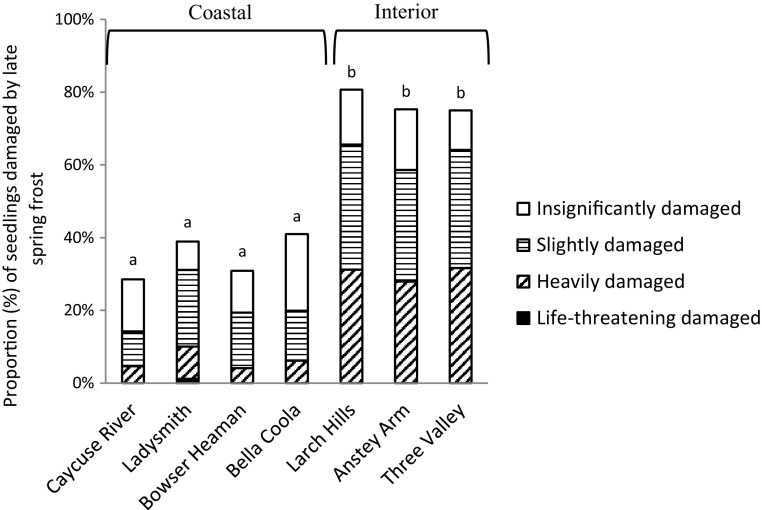
Proportion (%) of the seedlings damaged by late-spring frost in spring 2011. Means with the same letter indicate no significant differences at *p* = 0.05

**Fig. 5:**
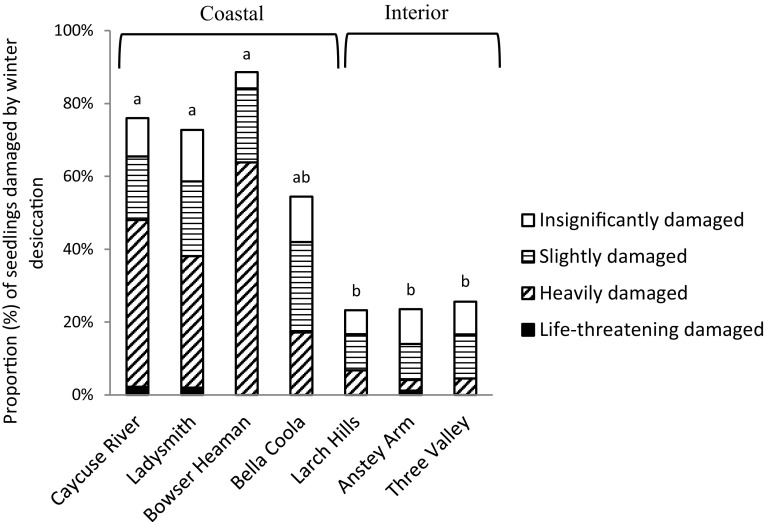
Proportion (%) of seedlings damaged by winter desiccation in spring 2013. Means with the same letter indicate no significant differences at *p* = 0.05

The interior variety were more frequently, and severely, damaged by late-spring frost in 2011 than the coastal variety (*p* < 0.0001) (Fig. 4). Similarly, in spring 2013, a high proportion of seedlings from the coastal variety (> 50%) were damaged by winter desiccation (Fig. 5). Causes of damage and death were difficult to identify after six growing seasons. Only damage caused by wild animals could be determined with sufficient certainty, but the damage affected only 2% of the seedlings on average.

Among seedlings of coastal origin that were undamaged in 2011, 42% were heavily damaged in 2013. Among heavily damaged seedlings in 2011, 50% were also heavily damaged in 2013. Corresponding proportions for the interior provenances were 10 and 4%, respectively (Table 2).

**Fig. 6:**
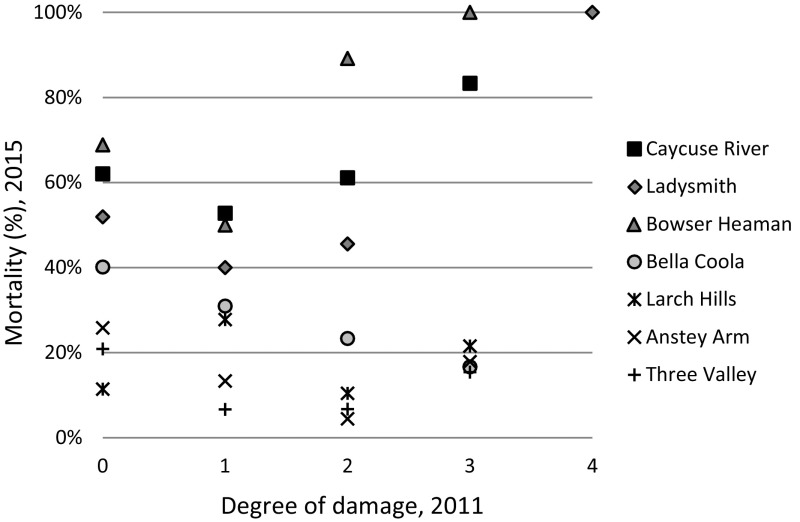
Mortality in 2015 (%) of seedlings registered as (0) undamaged, (1) insignificantly damaged, (2) slightly damaged, (3) heavily damaged and (4) life-threatening damaged by late-spring frost in 2011

Because the reason for seedling death could not be determined for the 2015 measurement, we followed each of the seedlings damaged by late-spring frost in 2011 and determined if the seedling was still alive in 2015. No pattern between late-spring frost damage and mortality was obvious (Fig. 6).

**Fig. 7:**
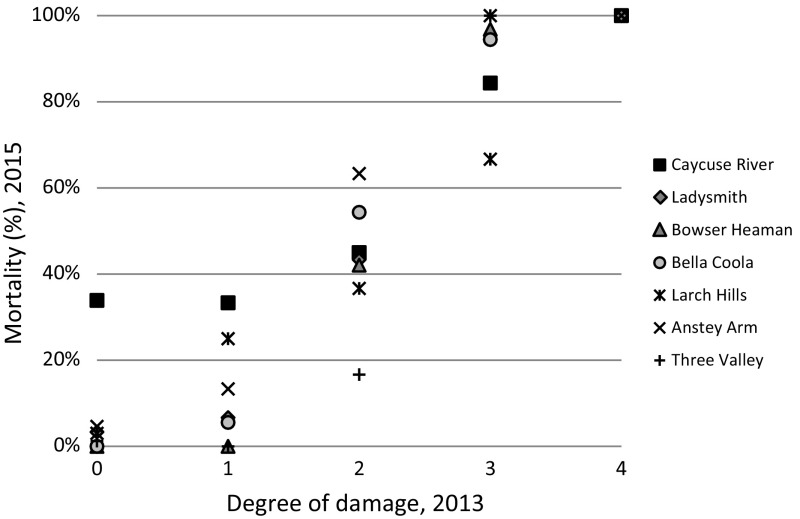
Mortality in 2015 (%) of seedlings registered as (0) undamaged, (1) insignificantly damaged, (2) slightly damaged, (3) heavily damaged and (4) life-threatening damaged by winter desiccation in spring 2013

Less than 10% of the seedlings that were heavily damaged by winter desiccation in spring 2013 were still alive in 2015 (Fig. 7). In addition, the mortality was high among the seedlings that were classified as slightly damaged in spring 2013. The mortality among the seedlings undamaged in 2013 was low, with the exception of the provenance Caycuse River. The damage in 2013 seems to have a strong connection with mortality in 2015.

### Timing of bud burst

**Fig. 8:**
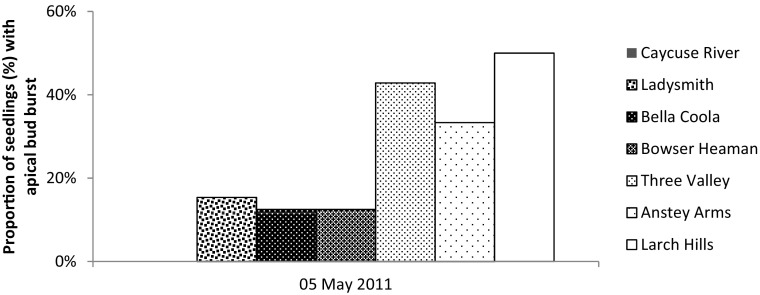
Proportion (%) of seedlings of each provenance that had burst the apical bud at time of the freezing event in May 5, 2011

The proportion of seedlings that had burst the apical bud at the time of the freezing event in May 2011, was higher for the interior variety (33–50%) than the coastal variety (0–15%) (Fig. 8). Proportion of seedlings that were heavily damaged by late-spring frost were 28–32% for the interior variety and 4–9% for the coastal variety (Fig. 4).

### Growth

Mean seedling height after six growing seasons ranged between 1.77 m for Caycuse River and 2.55 m for Bella Coola. However, no significant differences occurred among provenances (*p* = 0.36) or between the coastal and interior varieties (*p* = 0.93). The mean length of the leading shoot for the sixth growing season showed the same pattern; Caycuse River showed the shortest average length of leading shoot (0.47 m) and Bella Coola was the provenance with the longest average length of leading shoot (0.69 m). No significant differences among provenances (*p* = 0.45), or between the two varieties (*p* = 0.67) were observed.

## Discussion

### Survival

Survival of the coastal Douglas-fir provenances was low after six growing seasons (24–58%) compared with the interior provenances (73–78%). The provenances originated from latitudes between 48°N and 52°N. These results are in agreement with the earlier Swedish provenance trial at Tönnersjöheden experimental forest at age 9 years (Martinsson and Kollenmark 2001). In a provenance trial in the Netherlands, survival 4 years after planting ranged from 63 to 99% among 18 coastal provenances originating from latitudes between 44°N and 50°N (Eilmann et al. 2013). In Ireland, coastal provenances showed a higher survival rate than interior provenances from high elevations that suffered from late-spring frost damage (Lally and Thomson 1998). The same pattern of late-spring frost affecting the interior provenances was observed in the present study. However, it did not affect the survival. Instead, the interior Douglas-fir coped with the total frost damage, late-spring frost and winter desiccation, in a more successful way than the coastal Douglas-fir at this specific site. The climate in south-west of Sweden is regarded as maritime, due to, for Sweden, comparatively high mean annual temperature and high annual precipitation (Alexandersson and Eggertsson Carlström 2001). Compared to Vancouver Island, from which three of the coastal provenances originate, the winters in south-west Sweden would rather be considered as harsh and variable (Fig. 2) (Government of Canada 2018).

### Frost damage

In spring 2011, 1 year after planting, there was a period of very warm weather, followed by several nights with temperatures below zero. When chilling requirements have been met and dormancy is broken, temperatures higher than + 5 °C force bud burst (Bailey and Harrington 2006). Campbell and Sugano (1979) found that interior Douglas-fir had a lower chilling requirement than coastal Douglas-fir, and observed earlier bud burst in spring for the interior variety. A study performed by Malmqvist et al. (2017) with the same seed origin as in this study, showed the same pattern of earlier bud burst for the interior variety compared to the coastal variety. All interior provenances in this study had started the process of bud development at the time of the freezing temperatures mentioned above and were therefore damaged by frost to a higher extent than the late flushing coastal provenances. The severe late-spring frost was, however, not fatal. Although late-spring frost probably will occur periodically, the risk for serious damage diminishes with time as the seedlings grow taller. Trees taller than 1–2 m seldom suffer from damage by late-spring frosts while the apical shoot and upper branches are above the coldest air (Morén and Perttu 1994). Jönsson et al. (2004) and Langvall (2011) highlight the increased risk for late-spring frost damage in Norway spruce in southern Sweden due to climate change. This risk can be reduced with methods such as shelterwood, planting on slopes, and mechanical site preparation in order to reduce spring frost damage and increase seedling survival (Newsome et al. 1990; Langvall 2000).

Winter desiccation is thought to be the most serious type of damage appearing in winter and early spring (Christersson and von Fircks 1988). Such damage could limit the successful establishment of conifer seedlings (Sakai and Larcher 1987). In the present study, the climate conditions preceding winter desiccation (low night temperature, large short-term temperature fluctuations, and high irradiation) were present in spring 2013. Damage from winter desiccation affected the coastal variety more frequently and more severely than the interior variety. Christersson and von Fircks (1988) found differences in response to winter desiccation between pine and spruce species, but the cause for the difference was uncertain. In the present study there was, however, a clear difference observed between the two varieties within the same species. Coastal Douglas-fir are known to have a later growth cessation than interior Douglas-fir, due to adaption to the different climatic conditions in the areas from which they originate (Rehfeldt 1977). The delay could result in lower freezing tolerance. Malmqvist et al. 2016 showed that coastal Douglas-fir seedlings of the same origin as used in the present study developed freezing tolerance in roots and shoots later in the autumn than the interior Douglas-fir. If the roots of the seedlings in the present study were damaged during the autumn, this may have influenced how the winter desiccation later affected the seedlings, especially seedlings from the coastal areas. Bansal et al. (2015) drew attention to the risk that the environmental cues that trigger cold acclimation may disappear as the climate changes, resulting in delayed cold hardening. The mortality among the seedlings subjected to winter desiccation was found to be much higher than expected at the time of recording the damage. The seedlings judged as heavily damaged were nearly all dead 2 years later. The seedlings graded as slightly damaged (damage class 2), had a mortality of almost 50% after 2 years.

### Growth

In the present study, the early growth of the interior Douglas-fir was not significantly lower than of the coastal Douglas-fir after six growing seasons. Results from other countries in Europe, such as Germany, Bulgaria, and Denmark, imply that the interior Douglas-fir may not reach the same growth capacity as the more fast growing coastal Douglas-fir (Kleinschmit et al. 1974; Kleinschmit and Bastien 1992; Hansen et al. 2005; Petkova et al. 2014). In Sweden, however, the climate may not allow coastal Douglas-fir to thrive at full capacity, and extreme climate events may be more influential. With climate change, unseasonal climate events may increase in frequency, forcing us to reconsider which origin may be deemed suitable (Eilmann et al. 2013). In North America, as well as in several countries in Europe, new recommendations based on trials and models are still in progress, with greater specific consideration to drought tolerance and cold hardiness in addition to questions of growth capacity (Isaac-Renton et al. 2014; Bansal et al. 2015, 2016; Chakraborty et al. 2016). Isaac-Renton et al. (2014) suggested that the interior Douglas-fir are likely to be a more appropriate and safe choice for regeneration in continental climates in Finland and Central and Eastern Europe. The severity of the damage from winter desiccation among the coastal Douglas-fir in this study entails parts of Sweden to join that group. This study also highlights the need to reconsider the choice of variety and provenance in a changing climate.
